# Poster Session I - A72 COMPARATIVE OUTCOMES OF ANESTHESIA VS. ENDOSCOPIST-CONTROLLED SEDATION IN ERCP: A RETROSPECTIVE STUDY

**DOI:** 10.1093/jcag/gwaf042.072

**Published:** 2026-02-13

**Authors:** R Kakkar, B Dhaliwal, R Uy, N Johal, C Leung, J J Telford, R A Enns, D Motomura, E Lam

**Affiliations:** Internal Medicine, The University of British Columbia Faculty of Medicine, Vancouver, BC, Canada; Internal Medicine, The University of British Columbia Faculty of Medicine, Vancouver, BC, Canada; The University of British Columbia Faculty of Medicine, Vancouver, BC, Canada; Internal Medicine, The University of British Columbia Faculty of Medicine, Vancouver, BC, Canada; Internal Medicine, The University of British Columbia Faculty of Medicine, Vancouver, BC, Canada; Internal Medicine, The University of British Columbia Faculty of Medicine, Vancouver, BC, Canada; Internal Medicine, The University of British Columbia Faculty of Medicine, Vancouver, BC, Canada; Internal Medicine, The University of British Columbia Faculty of Medicine, Vancouver, BC, Canada; Internal Medicine, The University of British Columbia Faculty of Medicine, Vancouver, BC, Canada

## Abstract

**Background:**

Endoscopic retrograde cholangiopancreatography (ERCP) is a complex endoscopic intervention used to manage pancreaticobiliary disorders. Sedation is essential for procedural success, ranging from endoscopist-controlled conscious sedation (CS) to anesthesiologist-administered deep sedation or general anesthesia (GA). While anesthesia use in ERCP is increasing across North America, data comparing sedation strategies in Canadian practice remain limited. Prior studies suggest potential benefits of anesthesia-assisted sedation in reducing procedural failure but raise concerns about cost, resource use, and unclear impact on patient-centered outcomes.

**Aims:**

To compare patient characteristics, procedural outcomes, and sedation-related complications between ERCPs performed with endoscopist-controlled CS and those with anesthesia-assisted sedation at a large Canadian tertiary care center.

**Methods:**

This retrospective cohort study included ERCPs performed at St. Paul’s Hospital in Vancouver between August 2023 and July 2025. Patient demographics, sedation modality, procedural characteristics, and clinical outcomes were extracted through chart review. The primary outcome was sedation-related adverse events. Secondary outcomes included length of procedure, recovery time, post-procedural admission, and need for early repeat ERCP.

**Results:**

A total of 938 ERCPs were analyzed, with 809 under endoscopist and 129 under anesthesia-assisted sedation. Desaturation and hypotension were more common with anesthesia (desaturation 11% vs. 2%, p < 0.005; hypotension 19% vs. 3%, p < 0.0001). Overall sedation-related complication rates were low and comparable between groups (1% in both). The anesthesia group had longer procedures (mean 40 vs. 27 minutes, p < 0.0001), and more frequent use of advanced interventions including Spyglass (15% vs. 1%). Repeat ERCP within 8 weeks was more frequent in the anesthesia group (33% vs. 15%), with most patients receiving the same sedation type for repeat intervention.

**Conclusions:**

Anesthesia-assisted ERCP was associated with longer procedures, more therapeutic interventions, and higher rates of intra-procedural hypotension and desaturation, without an increase in overall complication rates. Notably, repeat ERCP within 8 weeks was more frequent in the anesthesia group, likely reflecting higher procedural complexity and planned staged interventions. These findings support the selective use of anesthesia for challenging ERCPs while highlighting the safety and efficiency of endoscopist-controlled sedation for most cases.

**Funding Agencies:**

None

